# Evaluating the impacts of previous breast surgery on surgical complications and aesthetic outcomes in reverse-sequence endoscopic-assisted nipple-sparing mastectomy with or without direct-to-implant breast reconstruction

**DOI:** 10.3389/fonc.2026.1860773

**Published:** 2026-09-09

**Authors:** Xiaoman Cao, Tao Zhu, Tianyuan Li, Zhongjian Zhu, Kawun Chung, Hui Dai, Qing Zhang, Yu Feng, Jiao Zhou, Huanzuo Yang, Donglin Zhang, Zhenggui Du

**Affiliations:** 1 Breast Centre, West China Hospital, Sichuan University, Chengdu, China; 2 Department of General Surgery, West China Hospital, Sichuan University, Chengdu, China; 3 Department of General Surgery, Fourth People’s Hospital of Sichuan Province, Chengdu, China

**Keywords:** breast cancer, direct-to-implant breast reconstruction, previous breast surgery, reverse-sequence endoscopic-assisted nipple-sparing mastectomy, surgical complications

## Abstract

**Purpose:**

The reverse-sequence endoscopic-assisted nipple-sparing mastectomy (R-E-NSM), with or without direct-to-implant breast reconstruction (DTI-BR), has become increasingly popular in China. This study aimed to evaluate whether previous breast surgery (PBS) affects surgical complications and aesthetic outcomes after R-E-NSM.

**Methods:**

Clinical data of patients who underwent R-E-NSM at the Breast Center, West China Hospital, Sichuan University, between April 30, 2020, and April 30, 2025, were prospectively collected and analyzed.

**Results:**

A total of 1081 patients were enrolled in the study. Among them, 89 had intraoperative breast surgery (IBS), 56 had short-term previous breast surgery (SPBS), 75 had long-term previous breast surgery (LPBS), and 861 had no previous breast surgery (NPBS). SPBS and IBS were not significantly associated with increased complication risk after multivariable adjustment (all P > 0.05). LPBS was significantly associated with lower odds of any complications (OR, 0.238; 95% CI, 0.089–0.633; P = 0.040), minor complications (OR, 0.304; 95% CI, 0.122–0.752; P = 0.010), and CDC ≥ 2 complications (OR, 0.333; 95% CI, 0.125–0.885; P = 0.027). BREAST-Q scores did not differ significantly among groups. Compared with the NPBS group, the SPBS group showed a statistically significant difference in Ueda scores at 3 months (P = 0.014). Oncologic outcomes did not differ significantly among the four groups (all P > 0.05).

**Conclusion:**

For patients with SPBS, implementing R-E-NSM warrants careful clinical evaluation due to potential safety concerns. Among patients with LPBS, current evidence indicates that incorporating R-E-NSM is not an independent risk factor and may be linked to a significantly lower incidence of complications.

## 1 Introduction

Breast cancer is the most common malignancy among women and ranks among the top three cancers globally (1). From radical mastectomy to breast-conserving surgery, surgical intervention remains the cornerstone of breast cancer treatment. Recently, endoscopic-assisted nipple-sparing mastectomy (E-NSM) and direct-to-implant breast reconstruction (DTI-BR) have seen increased adoption utilization (2–4). Numerous studies have demonstrated the feasibility, safety, discreet incision, and favorable aesthetic results of E-NSM (3, 5–10). However, E-NSM faces several challenges. These include high technical difficulty, a long learning curve, and extended operation time, mainly due to difficulties in establishing an adequate operating space and the potential for instrument interference (11–13).

Our team proposed reverse-sequence endoscopic-assisted nipple-sparing mastectomy (R-E-NSM) with or without DTI-BR (14, 15). This technique uses the expanding power of gas to form a universal retractor and features an innovative reverse dissection sequence that progresses from deep to superficial layers. These improvements have been successful in boosting surgical efficiency and lowering the rate of complications compared to conventional NSM. Additionally, R-E-NSM has been performed safely at our day surgery center (14, 16). It addresses the traditional challenges that have hindered the progress of endoscopic breast reconstruction.

Core needle biopsy is currently the preferred diagnostic method for suspicious breast lesions and is considered the ‘gold standard’ for diagnosing breast cancer (17, 18). However, some patients in our cohort had undergone excisional biopsy at outside institutions prior to referral to our center. These patients can be categorized into several scenarios. First, some patients are unable to undergo needle biopsy. For example, when ductoscopy reveals an intraductal papilloma, these patients cannot undergo needle biopsy or minimally invasive surgery and must undergo excision of the diseased duct. Second, some patients had previously undergone surgery for benign masses before the current procedure. Third, although excisional biopsy is now rarely performed, some primary-level hospitals in China lack the capacity for needle biopsy or vacuum-assisted biopsy; therefore, such patients undergo excisional biopsy. We observed that some patients who underwent R-E-NSM needed removal of the skin of the nipple-areolar complex (NAC) or the tumor surface. These prior surgical interventions may influence the safety and aesthetic outcomes of subsequent R-E-NSM. To date, no studies have examined the impact of previous breast surgery (PBS) on surgical complications in patients undergoing R-E-NSM. Therefore, we conducted a prospective cohort study to determine whether PBS affects surgical complications and aesthetic outcomes in patients undergoing R-E-NSM with or without DTI-BR.

## 2 Methods

### 2.1 Ethical Statement

The study received approval from the Biomedical Ethics Committee of West China Hospital and was registered with the Chinese Clinical Trial Registry (ChiCTR2100047862). All participants provided written informed consent.

### 2.2 Study design and patients

The Breast Disease Center at West China Hospital, Sichuan University—one of China’s largest tertiary referral centers for breast cancer—treats approximately 2, 000 newly diagnosed breast cancer patients each year and maintains a prospective, institutional database (19). All consecutive patients who underwent R-E-NSM, with or without DTI-BR, at our center between April 30, 2020, and April 30, 2025, were prospectively enrolled. A flowchart outlining the data selection process is presented in **Supplementary Figure 1**.

A total of 1081 patients who underwent R-E-NSM with or without DTI-BR at our breast center between April 2020 and April 2025 were consecutively included in this study. We performed Receiver-operating characteristic (ROC) curve analysis to determine the optimal time interval from previous breast surgery to R-E-NSM that best predicts the occurrence of any complications. Only patients with a history of previous breast surgery (n=220, combining IBS, SPBS, and LPBS groups) were included. Patients without previous surgery (NPBS, n=861) were excluded as they had no prior surgical interval to measure. The optimal cutoff of 62 days was determined by ROC curve analysis based on the occurrence of any surgical complications, using the Youden index to maximize both sensitivity and specificity. The area under the curve (AUC) was 0.586 (95% CI: 0.493–0.680), with a sensitivity of 36.8% and specificity of 87.5% (**Supplementary Figure 2**). Patients were divided into four groups: intraoperative breast surgery (IBS) (excision of the skin or NAC during R-E-NSM, but no previous breast surgery), short-term previous breast surgery (SPBS) (previous breast surgery within 62 days), long-term previous breast surgery (LPBS) (previous breast surgery more than 62 days ago), and no previous breast surgery (NPBS). There were 89 patients (8.2%) in the IBS group, 56 patients (5.2%) in the SPBS group, 75 patients (6.4%) in the LPBS group, and 861 patients (79.2%) in the NPBS group.

### 2.3 Indications of R-E-NSM

Eligible breast cancer patients had tumors no larger than 5 cm, clinical nodal stage cN0–cN2, with no evidence of skin or chest wall infiltration, and no clinical or radiographic signs of distant metastasis. Prophylactic mastectomy was performed in women with BRCA1/2 mutations, high-risk lesions, or those who desired a risk-reducing mastectomy following risk counseling. Patients were excluded if tumors exceeded size limits, involved the nipple–areolar complex or pectoralis major, showed extensive skin infiltration, had fixed axillary nodes, or if severe comorbidities (cardiac, hepatic, renal insufficiency, or poor performance status) prevented safe surgery.

### 2.4 Surgical procedure

The details of the surgical technique for R-E-NSM and its combination with various breast reconstruction techniques have been described in our previous reports (15, 20, 21). Briefly, R-E-NSM utilizes CO_2_ insufflation to create an endoscopic working space and employs a reverse dissection sequence (deep-to-superficial) through a single axillary incision. Key steps include axillary lymph node management, dissociation of the subpectoral or prepectoral envelope, endoscopic dissociation of subcutaneous glandular tissue, and direct-to-implant reconstruction. For patients requiring intraoperative breast surgery, additional procedures such as NAC resection or tumor surface skin excision were performed as clinically indicated.

### 2.5 Outcome evaluation

All surgical complications and aesthetic outcomes were recorded prospectively during standardized outpatient visits and independently verified by the principal investigator to ensure data completeness and accuracy.

Surgical complications included hematoma/hemorrhage, surgical site infection (SSI), delayed incision healing/dehiscence, seroma, skin flap/NAC ischemia/necrosis, which was defined and graded with the Clavien–Dindo classification (CDC) as minor complications (CDC grade I–II) or major complications (CDC grade III above) (22–24).

We assessed satisfaction with breast outcomes, as well as psychosocial, physical, and sexual well-being, using the BREAST-Q questionnaire (25). The BREAST-Q is commonly used to assess health-related satisfaction and quality of life in patients who have undergone breast reconstruction after mastectomy. Responses for each subscale are scored from 0 to 100, with higher scores indicating better outcomes. Patients completed the online questionnaire before mastectomy reconstruction and at 6 months after reconstruction. For the doctor-reported cosmetic outcomes, the Ueda scoring system was used to evaluate both preoperative and postoperative photographs (26).

Local recurrence-free survival (LRFS), distant metastasis-free survival (DMFS), and disease-free survival (DFS) were used as the primary study endpoints. LRFS was defined as the time from the surgery date to recurrence in the ipsilateral chest wall or at the breast surgical site. DMFS was defined as the time from surgery to recurrence at distant sites. DFS was defined as the time from the surgery date to recurrence, metastasis, new breast cancer on the same or opposite side, and death from any cause.

### 2.6 Statistical analysis

Categorical variables were compared using the χ2 test. Continuous variables were compared using a t-test. Receiver operating characteristic (ROC) curves were used to predict time points and categorize patients into four groups. Complication outcomes were analyzed using univariate logistic regression to obtain adjusted odds ratios (ORs) with 95% confidence intervals (CIs). The mixed-effects logistic regression model was employed to analyze the impact of PBS on complications and cosmetic outcomes. Survival curves were estimated by the Kaplan–Meier method for LRFS, DMFS, and DFS, and the log-rank test was used for between-group comparisons. A p-value less than 0.05 was considered significant, and all tests were two-tailed. All data analyses were performed using Statistical Product and Service Solutions (SPSS) version 26.0.0.0 (IBM Corporation; Armonk, New York, USA).

## 3 Results

### 3.1 Baseline characteristics

The patient demographic and clinical characteristics by PBS are shown in **Table 1**. During the study period, the median (IQR) follow-up was 31.1 (17.1–46.2) months [29.9 (16.6–43.6) months for NPBS, 47.6 (31.9–54.2) months for IBS, 34.0 (15.5–45.0) months for SPBS, and 27.0 (17.0–42.1) months for LPBS]. The mean (SD) age was 42.0 ± 8.9, 40.9 ± 7.8, 37.7 ± 8.6, and 41.4 ± 8.9 years in the NPBS, IBS, SPBS, and LPBS groups (P = 0.005). The median (IQR) operation times were 146.0 (120.0–190.0), 177.5 (160.8–230.0), 174.0 (141.8–210.0), and 159.5 (123.5–201.8) minutes for the NPBS, IBS, SPBS, and LPBS groups (P<0.001). Additionally, among the 220 patients undergoing intraoperative and previous breast surgery, the most common incision was the periareolar arc, observed in 130 patients (59.1%) (**Supplementary Table 1**).

**Table 1:** Demographic and clinical characteristics of the patients.

Characteristic	NPBS (n=861)	IBS (n=89)	SPBS (n=56)	LPBS (n=75)	P value
Age, Mean ± SD	42.0 ± 8.9	40.9 ± 7.8	37.7 ± 8.6	41.4 ± 8.9	0.005
BMI, Mean ± SD	22.2 ± 7.4	22.2 ± 3.1	22.1 ± 3.1	22.1 ± 2.9	1.000
Diabetes, *n* (%)					0.620
NA	4 (0.5)	0 (0.0)	0 (0.0)	0 (0.0)	
No	844 (98.0)	86 (96.6)	56 (100.0)	73 (97.3)	
Yes	13 (1.5)	3 (3.4)	0 (0.0)	2 (2.7)	
Hypertension, *n* (%)					0.952
NA	4 (0.5)	0 (0.0)	0 (0.0)	0 (0.0)	
No	824 (95.7)	85 (95.5)	55 (98.2)	73 (97.3)	
Yes	33 (3.8)	4 (4.5)	1 (1.8)	2 (2.7)	
Drinking, *n* (%)					0.837
NA	5 (0.6)	0 (0.0)	0 (0.0)	0 (0.0)	
No	846 (98.3)	87 (97.8)	56 (100.0)	74 (98.7)	
Yes	10 (1.2)	2 (2.2)	0 (0.0)	1 (1.3)	
Smoking, *n* (%)					0.471
NA	5 (0.6)	0 (0.0)	0 (0.0)	0 (0.0)	
No	847 (98.4)	86 (96.6)	55 (98.2)	74 (98.7)	
Yes	9 (1.0)	3 (3.4)	1 (1.8)	1 (1.3)	
Cup size, *n* (%)					0.211
NA	20 (2.3)	0 (0.0)	3 (5.4)	1 (1.3)	
≤A	271 (31.5)	20 (22.5)	16 (28.6)	24 (32.0)	
B	358 (41.6)	44 (49.4)	27 (48.2)	34 (45.3)	
≥C	212 (24.6)	25 (28.1)	10 (17.9)	16 (21.3)	
Breast ptosis, *n* (%)					0.012
NA	29 (3.4)	0 (0.0)	4 (7.1)	1 (1.3)	
No	465 (54.0)	45 (50.6)	38 (67.9)	45 (60.0)	
I°	183 (21.3)	25 (28.1)	8 (14.3)	22 (29.3)	
II°	121 (14.1)	11 (12.4)	3 (5.4)	6 (8.0)	
III°	33 (3.8)	7 (7.9)	2 (3.6)	1 (1.3)	
Pseudoptosis	30 (3.5)	1 (1.1)	1 (1.8)	0 (0.0)	
Laterality, *n* (%)					0.287
NA	7 (0.8)	0 (0.0)	0 (0.0)	0 (0.0)	
Unilateral	664 (77.1)	66 (74.2)	43 (76.8)	49 (65.3)	
Bilateral	190 (22.1)	23 (25.8)	13 (23.2)	26 (34.7)	
Pathologic T stage ^a^, *n* (%)					<0.001
NA	61 (7.1)	5 (5.6)	6 (10.7)	14 (18.7)	
Tis	128 (14.9)	18 (20.2)	15 (26.8)	15 (20.0)	
T1	332 (38.6)	30 (33.7)	20 (35.7)	36 (48.0)	
≥T2	340 (39.5)	36 (40.4)	15 (26.8)	10 (13.3)	
Pathologic N stage ^a^, *n* (%)					0.001
NA	49 (5.7)	7 (7.9)	3 (5.4)	12 (16.0)	
N0	600 (69.7)	56 (62.9)	47 (83.9)	55 (73.3)	
N1	165 (19.2)	22 (24.7)	6 (10.7)	4 (5.3)	
≥N2	47 (5.5)	4 (4.5)	0 (0.0)	4 (5.3)	
ER Positive ^a^, *n* (%)					0.116
NA	84 (9.8)	7 (7.9)	8 (14.3)	15 (20.0)	
Negative	204 (23.7)	24 (27.0)	12 (21.4)	13 (17.3)	
Positive	573 (66.6)	58 (65.2)	36 (64.3)	47 (62.7)	
PR Positive ^a^, *n* (%)					0.014
NA	94 (10.9)	7 (7.9)	11 (19.6)	18 (24.0)	
Negative	222 (25.8)	25 (28.1)	12 (21.4)	18 (24.0)	
Positive	545 (63.3)	57 (64.0)	33 (58.9)	39 (52.0)	
Her-2 Positive ^a^, *n* (%)					0.065
NA	213 (24.7)	16 (18.0)	12 (21.4)	25 (33.3)	
Negative ^b^	455 (52.8)	49 (55.1)	37 (66.1)	40 (53.3)	
Positive	193 (22.4)	24 (27.0)	7 (12.5)	10 (13.3)	
Ki-67 ^a^, *n* (%)					0.012
NA	120 (13.9)	10 (11.2)	11 (19.6)	22 (29.3)	
≤20%	379 (44.0)	44 (49.4)	22 (39.3)	32 (42.7)	
>20%	362 (42.0)	35 (39.3)	23 (41.1)	21 (28.0)	
Neoadjuvant chemotherapy, *n* (%)					<0.001
NA	10 (1.2)	1 (1.1)	0 (0.0)	2 (2.7)	
No	667 (77.5)	70 (78.7)	56 (100.0)	65 (86.7)	
Yes	184 (21.4)	18 (20.2)	0 (0.0)	8 (10.7)	
Operation time, M (Q_1_, Q_3_)	146.0 (120.0, 190.0)	177.5 (160.8, 230.0)	174.0 (141.8, 210.0)	159.5 (123.5, 201.8)	<0.001
Operation type, *n* (%)					<0.001
PBR	424 (49.2)	36 (40.4)	22 (39.3)	38 (50.7)	
Dual-plan BR	320 (37.2)	23 (25.8)	24 (42.9)	18 (24.0)	
SBR	73 (8.5)	23 (25.8)	5 (8.9)	13 (17.3)	
LD with/without implant	34 (3.9)	7 (7.9)	4 (7.1)	5 (6.7)	
NSM	10 (1.2)	0 (0.0)	1 (1.8)	1 (1.3)	
Axillary lymph node management, *n* (%)					<0.001
NA	22 (2.6)	1 (1.1)	0 (0.0)	4 (5.3)	
Untreated	32 (3.7)	3 (3.4)	7 (12.5)	11 (14.7)	
SLNB	558 (64.8)	53 (59.6)	41 (73.2)	48 (64.0)	
ALND	171 (19.9)	22 (24.7)	0 (0.0)	8 (10.7)	
ALND+SLNB	77 (8.9)	10 (11.2)	8 (14.3)	4 (5.3)	
Nipple resection, *n* (%)					<0.001
NA	6 (0.7)	0 (0.0)	0 (0.0)	0 (0.0)	
No	855 (99.3)	11 (12.4)	56 (100.0)	75 (100.0)	
Yes	0 (0.0)	78 (87.6)	0 (0.0)	0 (0.0)	
Nipple reconstruction, *n* (%)					<0.001
NA	38 (4.4)	6 (6.7)	3 (5.4)	4 (5.3)	
No	821 (95.4)	34 (38.2)	53 (94.6)	71 (94.7)	
Yes	2 (0.2)	49 (55.1)	0 (0.0)	0 (0.0)	
Mastectomy weight ^c^, g, M (Q_1_, Q_3_)	351.0 (264.5, 463.8)	294.9 (251.8, 455.0)	317.5 (230.5, 411.2)	333.0 (260.0, 464.0)	0.491
Implant size ^d^, cc, M (Q_1_, Q_3_)	305.0 (245.0, 355.0)	280.0 (245.0, 350.0)	315.0 (245.0, 347.5)	315.0 (245.0, 355.0)	0.423
Hospitalization, *n* (%)					0.134
NA	9 (1.0)	0 (0.0)	1 (1.8)	0 (0.0)	
Day surgery	433 (50.3)	35 (39.3)	31 (55.4)	31 (41.3)	
Inpatient surgery	419 (48.7)	54 (60.7)	24 (42.9)	44 (58.7)	
Hospital stays, days, M (Q_1_, Q_3_)	1.0 (1.0, 6.0)	6.0 (1.0, 7.0)	1.0 (1.0, 6.0)	5.0 (1.0, 6.0)	<0.001
Adjuvant chemotherapy, *n* (%)					0.060
NA	62 (7.2)	0 (0.0)	3 (5.4)	5 (6.7)	
No	407 (47.3)	43 (48.3)	29 (51.8)	42 (56.0)	
Yes	392 (45.5)	46 (51.7)	24 (42.9)	28 (37.3)	
Radiotherapy, *n* (%)					0.162
NA	123 (14.3)	12 (13.5)	8 (14.3)	9 (12.0)	
No	552 (64.1)	51 (57.3)	42 (75.0)	54 (72.0)	
Yes	186 (21.6)	26 (29.2)	6 (10.7)	12 (16.0)	
Adjuvant endocrinotherapy, *n* (%)					0.538
NA	131 (15.2)	7 (7.9)	8 (14.3)	8 (10.7)	
No	235 (27.3)	27 (30.3)	14 (25.0)	24 (32.0)	
Yes	495 (57.5)	55 (61.8)	34 (60.7)	43 (57.3)	
Anti-Her2 therapy, *n* (%)					0.039
NA	151 (17.5)	8 (9.0)	12 (21.4)	11 (14.7)	
No	552 (64.1)	57 (64.0)	40 (71.4)	53 (70.7)	
Yes	158 (18.4)	24 (27.0)	4 (7.1)	11 (14.7)	
Follow-up time, months, M (Q_1_, Q_3_)	29.9 (16.6, 43.6)	47.6 (31.9, 54.2)	34.0 (15.5, 45.0)	27.0 (17.0, 42.1)	<0.001

IBS, intraoperative breast surgery; SPBS, short-term previous breast surgery; LPBS, long-term previous breast surgery; NPBS, no previous breast surgery; M, median; SD, standard deviation; IQR, interquartile range; NA, not available; BMI, body mass index; ER, estrogen receptor; PR, progesterone receptor; HER-2, human epidermal growth factor receptor 2; PBR, prepectoral breast reconstruction; Dual-plan BR, Dual-plan breast reconstruction; SBR, subpectoral breast reconstruction; LD, latissimus dorsi; NSM, nipple-sparing mastectomy; SLNB, sentinel lymph node biopsy; ALND, axillary lymph node dissection.

^a^ Only including malignant cases.

^b^ Including carcinoma *in situ*.

^c^ There were a total of 1333 breasts. NA = 820. The NPBS group had 414 breasts, the IBS group had 40 breasts, the SPBS group had 26 breasts, and the LPBS group had 33 breasts.

^d^ There were a total of 1333 breasts. NA = 2. The NPBS group had 1049 breasts, the IBS group had 112 breasts, the SPBS group had 69 breasts, and the LPBS group had 101 breasts.

### 3.2 Surgical complications

The complication rates for each group are shown in **Table 2**. Any complications (P = 0.009), minor complications (P = 0.021), and CDC ≥ 2 complications (P = 0.023) varied significantly among the four groups. Specifically, the SPBS group had higher rates of any complications (28.6%), minor complications (25.0%), and CDC ≥ 2 complications (23.2%) compared to the other three groups. The LPBS group had a lower rate of any complication (6.7%) than the other groups. Among these, the incidence of incision dehiscence (3.6%) as a major complication was higher in the SPBS group (P = 0.044).

**Table 2:** Comparison of the surgical complications in the IBS, SPBS, LPBS, and NPBS groups.

Variable	NPBS (n=861)	IBS (n=89)	SPBS (n=56)	LPBS (n=75)	P value
Any complication	189 (22.0)	19 (21.3)	16 (28.6)	5 (6.7)	0.009
Major complications (CDC ≥ IIIa)	21 (2.4)	4 (4.5)	3 (5.4)	0 (0.0)	0.117
Implant loss	13 (1.5)	2 (2.2)	2 (3.6)	0 (0.0)	0.289
Hematoma/Hemorrhage (reoperation)	1 (0.1)	0 (0.0)	0 (0.0)	0 (0.0)	1.000
Flap necrosis (reoperation)	9 (1.0)	0 (0.0)	0 (0.0)	0 (0.0)	1.000
NAC ischemia (reoperation)	5 (0.6)	0 (0.0)	0 (0.0)	0 (0.0)	1.000
Wound dehiscence (reoperation)	5 (0.6)	2 (2.2)	2 (3.6)	0 (0.0)	0.044
SSI (reoperation)	4 (0.5)	1 (1.1)	1 (1.8)	0 (0.0)	0.284
Minor complications (CDC I-II)	180 (20.9)	17 (19.1)	14 (25.0)	5 (6.7)	0.021
Hematoma/Hemorrhage	31 (3.6)	3 (3.4)	1 (1.8)	1 (1.3)	0.827
SSI	118 (13.7)	14 (15.7)	8 (14.3)	4 (5.3)	0.191
Wound dehiscence	11 (1.3)	0 (0.0)	4 (7.1)	0 (0.0)	0.016
Flap ischemia	15 (1.7)	2 (2.2)	1 (1.8)	0 (0.0)	0.672
NAC ischemia	32 (3.7)	2 (2.2)	1 (1.8)	1 (1.3)	0.789
CDC ≥ II, N (%)	146 (17.0)	18 (20.2)	13 (23.2)	4 (5.3)	0.023
Repeated SSI, N (%)	85 (9.9)	13 (14.6)	8 (14.3)	2 (2.7)	0.052

IBS, intraoperative breast surgery; SPBS, short-term previous breast surgery; LPBS, long-term previous breast surgery; NPBS, no previous breast surgery; NAC, Nipple-Areolar Complex; CDC, Clavien–Dindo classification; SSI, surgical site infection.

**Figure 1:**
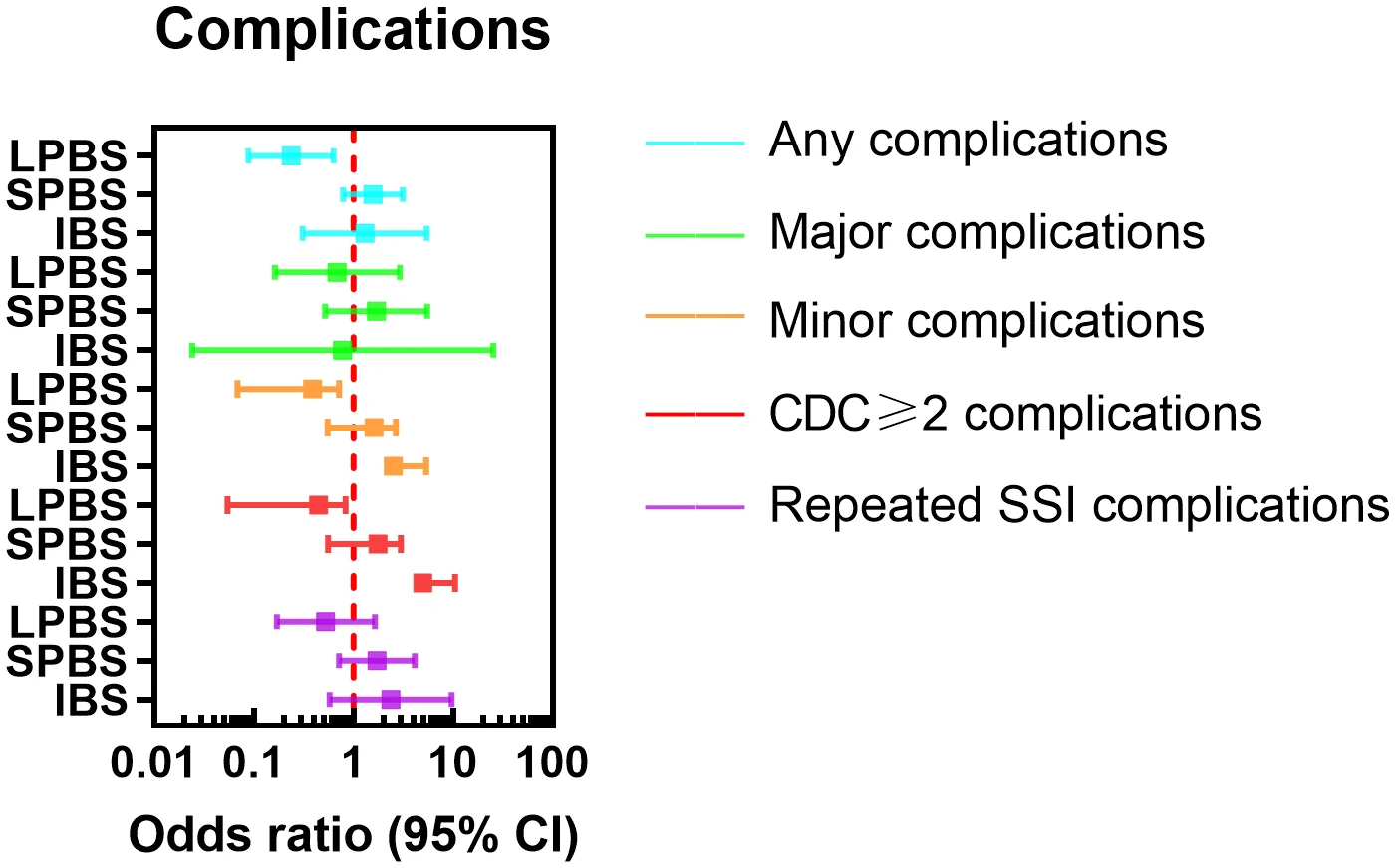
The forest plot of surgical complications. CDC, Clavien–Dindo classification, SSI, surgical site infection.

To reduce the influence of baseline differences on the comparison between groups, we conducted a mixed-effects model analysis. In the mixed-effects regression model, there were no statistically significant differences in surgical complications between the IBS and NPBS groups across all outcome measures (all P > 0.05). Similarly, SPBS was not significantly associated with increased odds of any, major, minor, CDC ≥2, or repeated SSI complications compared with NPBS (all P > 0.05). A trend toward higher odds of any complications was observed (OR 1.579; 95% CI 0.791–3.155; P = 0.195). In contrast, LPBS was significantly associated with lower odds of any complications (OR 0.238; 95% CI 0.089–0.633; P = 0.040), minor complications (OR 0.304; 95% CI 0.122–0.752; P = 0.010), and CDC ≥2 complications (OR 0.333; 95% CI 0.125–0.885; P = 0.027) compared with the NPBS group. No significant association was observed between LPBS and major complications or repeated SSI (both P > 0.05). (**Table 3**). The forest plots visually represented the significant results (**Figure 1**).

**Table 3:** Mixed-effect regression model of the surgical complications in the IBS, SPBS, LPBS, and NPBS groups.

	Any complication		Major complication		Minor complication		CDC≥2		Repeated SSI	
	OR	P	OR	P	OR	P	OR	P	OR	P
NPBS(n=861)	Ref	NA	Ref	NA	Ref	NA	Ref	NA	Ref	NA
IBS(n=89)	1.306 (0.311-5.491)	0.716	0.783 (0.024-25.496)	0.890	1.432 (0.353-5.806)	0.615	3.024 (0.817-11.193)	0.097	2.375 (0.580-9.720)	0.229
SPBS(n=56)	1.579 (0.791-3.155)	0.195	1.701 (0.520-5.558)	0.379	1.376 (0.687-2.758)	0.367	1.500 (0.724-3.107)	0.275	1.721 (0.717-4.131)	0.224
LPBS(n=75)	0.238 (0.089-0.633)	0.04	0.692 (0.163-2.930)	0.617	0.304 (0.122-0.752)	0.010	0.333 (0.125-0.885)	0.027	0.530 (0.171-1.647)	0.272

IBS, intraoperative breast surgery; SPBS, short-term previous breast surgery; LPBS, long-term previous breast surgery; NPBS, no previous breast surgery; CDC, Clavien–Dindo classification; SSI, surgical site infection; Ref, reference; NA, not available; OR, odds ratios; CI, confidence intervals.

Mixed-Effect Regression Model adjusted OR (95%CI): adjust for age, BMI, cup, breast ptosis, pathologic T stage, pathologic N stage, neoadjuvant chemotherapy, operation time, operation type, laterality, axillary lymph node management, nipple resection, nipple reconstruction, adjuvant chemotherapy, radiotherapy, and follow-up time.

### 3.3 Incision-related effects on complications

Concurrent with our analysis of complication rates, we evaluated the effect of incision-related factors. Missing data for incision-related variables (14.1%) were handled using a complete-case analysis (listwise deletion), retaining 189 of 220 patients (85.9%) with previous or intraoperative breast surgery for this exploratory analysis. Mixed-effects models showed no statistically significant differences in complications by incision location, laterality, or length in the overall cohort (all P > 0.05). However, we acknowledge that rare incision types (e.g., inferior fold incision, n=2) were underpowered for reliable inference, and wide confidence intervals reflect this uncertainty (**Supplementary Table 2**). Conclusions regarding incision-related effects are therefore primarily based on the more common incision types, particularly the periareolar arc incision (n=130).

### 3.4 Aesthetics outcomes

**Figure 2:**
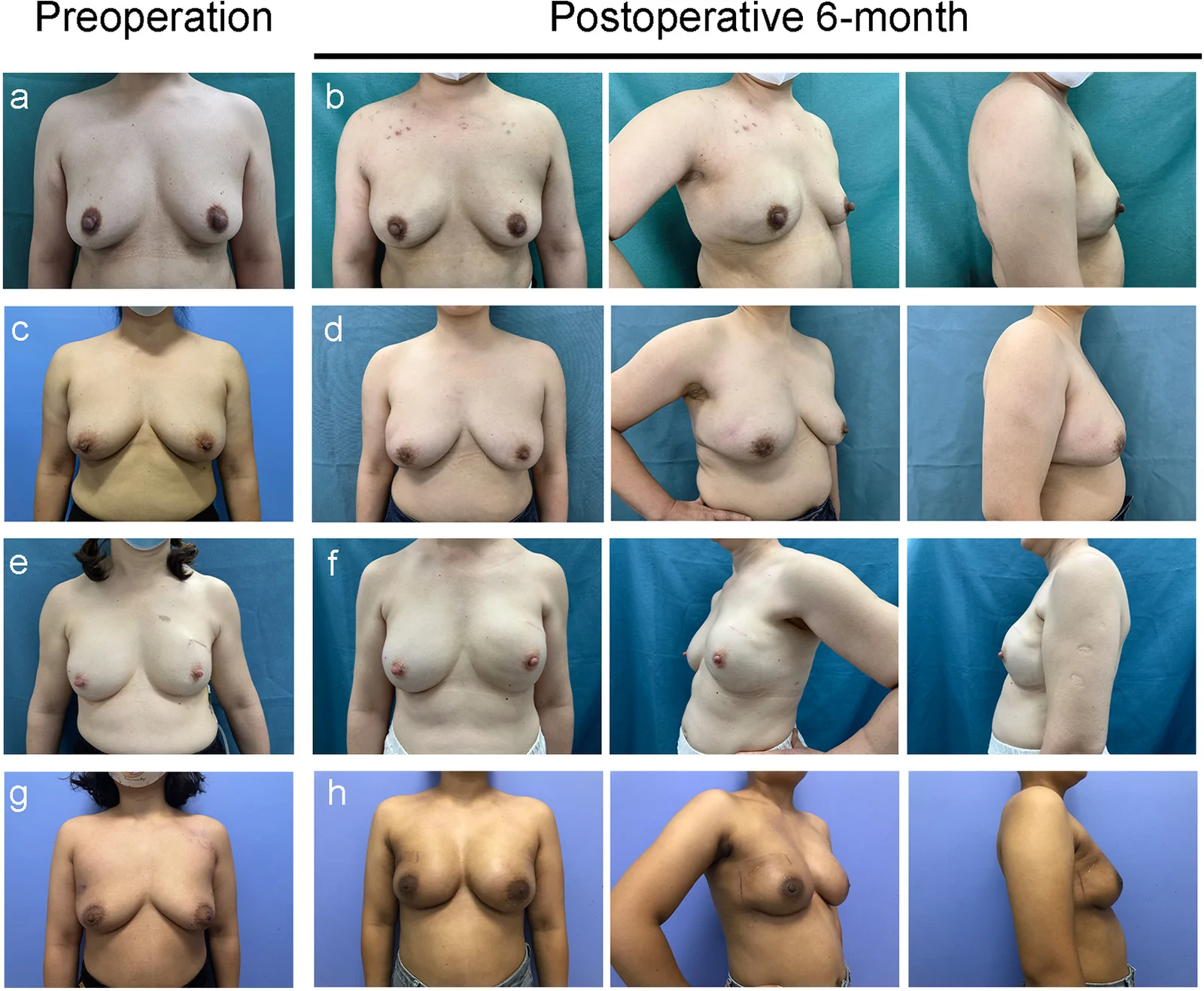
The preoperative and postoperative photos of the patients. **(A)** The preoperative and postoperative 6-month photos of the patients in the NPBS group; **(B)** The preoperative and postoperative 6-month photos of the patients in the IBS group; **(C)** The preoperative and postoperative 6-month photos of the patients in the SPBS group; **(D)** The preoperative and postoperative 6-month photos of the patients in the LPBS group. IBS, intraoperative breast surgery; SPBS, short-term previous breast surgery; LPBS, long-term previous breast surgery; NPBS, no previous breast surgery.

Patient-reported outcomes (BREAST-Q) and physician-reported aesthetic outcomes (Ueda scale) are summarized in **Table 4**. BREAST-Q scores did not differ significantly among the four groups at the postoperative time point. In contrast, Ueda scores showed significant differences between groups at 1 month (P < 0.001) and 3 months (P = 0.043) after surgery, with the highest proportion of “excellent” ratings in the LPBS group. The results from the mixed-effects regression model of Breast-Q scores are shown in **Supplementary Table 3**. There were no significant differences in satisfaction with their breasts, psychosocial well-being, physical well-being, or sexual well-being at 6 months among the four groups. The results from the mixed-effects regression model of Ueda scores are shown in **Supplementary Table 4**. Compared with the NPBS group, the SPBS group showed a statistically significant difference in Ueda scores at 3 months (P = 0.014). The preoperative and postoperative photos of the patients are displayed in **Figure 2**.

**Table 4:** Summary of patient-reported outcome and physician-reported outcome in the IBS, SPBS, LPBS, and NPBS groups.

		NPBS	IBS	SPBS	LPBS	P value
Patient-reported outcome on BREAST-Q, M (Q_1_, Q_3_)						
Satisfaction with breast	Preoperatively ^a^	58.0 (53.0, 64.0)	58.0 (53.0, 64.0)	58.0 (58.0, 64.0)	58.0 (48.0, 58.0)	0.151
	Postoperative 6 months ^b^	58.0 (53.0, 71.0)	60.0 (58.0, 70.5)	58.5 (55.0, 69.5)	59.0 (58.0, 75.0)	0.699
Psychosocial well-being	Preoperatively ^a^	74.0 (62.0, 92.0)	66.0 (58.0, 93.0)	77.0 (64.0, 87.0)	66.0 (60.0, 78.5)	0.223
	Postoperative 6 months ^b^	66.0 (60.0, 93.0)	74.0 (61.0, 98.5)	71.5 (64.0, 100.0)	66.0 (55.0, 87.0)	0.739
Physical well-being	Preoperatively ^a^	76.0 (62.0, 92.0)	76.0 (55.0, 92.0)	80.0 (76.0, 92.0)	68.0 (60.0, 82.5)	0.115
	Postoperative 6 months ^b^	64.0 (54.0, 76.0)	64.0 (55.0, 78.0)	66.0 (58.8, 76.8)	60.0 (50.0, 68.0)	0.502
Sexual well-being	Preoperatively ^a^	62.0 (48.0, 76.0)	59.5 (46.5, 74.0)	66.0 (58.2, 80.5)	56.0 (46.0, 66.0)	0.088
	Postoperative 6 months ^b^	53.0 (41.0, 66.0)	50.0 (46.5, 66.0)	66.0 (45.2, 73.5)	49.0 (41.0, 60.8)	0.339
Physician-reported outcome on Ueda, n (%)						
Postoperative 1-month ^c^	Excellent	635 (90.1)	37 (69.8)	35 (77.8)	52 (91.2)	<0.001
	Good	63 (8.9)	14 (26.4)	9 (20.0)	4 (7.0)	
	General	5 (0.7)	2 (3.8)	0 (0.0)	1 (1.8)	
	Poor	2 (0.3)	0 (0.0)	1 (2.2)	0 (0.0)	
Postoperative 3-month ^c^	Excellent	444 (82.1)	29 (72.5)	24 (68.6)	40 (90.9)	0.043
	Good	87 (16.1)	10 (25.0)	11 (31.4)	3 (6.8)	
	General	6 (1.1)	0 (0.0)	0 (0.0)	1 (2.3)	
	Poor	4 (0.7)	1 (2.5)	0 (0.0)	0 (0.0)	
Postoperative 6-month ^c^	Excellent	328 (74.7)	26 (78.8)	21 (84.0)	31 (86.1)	0.370
	Good	102 (23.2)	7 (21.2)	4 (16.0)	3 (8.3)	
	General	4 (0.9)	0 (0.0)	0 (0.0)	2 (5.6)	
	Poor	5 (1.1)	0 (0.0)	0 (0.0)	0 (0.0)	

IBS, intraoperative breast surgery; SPBS, short-term previous breast surgery; LPBS, long-term previous breast surgery; NPBS, no previous breast surgery; M, median.

^a^ Preoperative BREAST-Q score in satisfaction with breast NA = 376, preoperative BREAST-Q score in psychosocial well-being NA = 379, preoperative BREAST-Q score in psychosocial well-being NA = 379, preoperative BREAST-Q score in physical well-being NA = 382, preoperative BREAST-Q score in sexual well-being NA = 399, seven patients have no sexual life.

^b^ Postoperative 6 months BREAST-Q score in satisfaction with breast, NA = 670, postoperative 6 months BREAST-Q score in psychosocial well-being, NA = 668, postoperative 6 months BREAST-Q score in physical well-being, NA = 670, postoperative 6 months BREAST-Q score in sexual well-being, NA = 692, seven patients have no sexual life.

^c^ Ueda in postoperative 1 month NA = 221, Ueda in postoperative 3 months NA = 421, Ueda in postoperative 6 months NA = 548.

### 3.5 Oncologic outcomes

The median (IQR) follow-up time was 31.1 (17.1–46.2) months, 29.9 (16.6–43.6) months for NPBS, 47.6 (31.9–54.2) months for IBS, 34.0 (15.5–45.0) months for SPBS, and 27.0 (17.0–42.1) months for LPBS. The NPBS group had 11 cases of local recurrence and 7 cases of distant metastasis; the IBS group had 1 case of distant metastasis; the SPBS group had 1 case of local recurrence; and the LPBS group had 1 case of distant metastasis. The P values of LRFS, DMFS, and DFS were not significantly different among the four groups (**Supplementary Figure 3**).

## 4 Discussion

Currently, our team has published high-quality articles (3, 8–10, 14–16, 21, 27), and R-E-NSM with DTI-BR surgery has gained prominence. It has been demonstrated that R-E-NSM improves safety and enhances aesthetic outcomes (5–7, 28). However, during R-E-NSM, some patients might need removal of the skin of the NAC or the tumor surface. Patients who had an excisional biopsy before surgery may experience altered safety and aesthetic outcomes. This study found that IBS did not influence complication rates. However, the rates of any complication were higher in SPBS patients than in NPBS patients, and a trend toward higher odds of any complications was observed. Additionally, LPBS showed protective effects. Therefore, for patients with SPBS, the implementation of R-E-NSM should be done with caution. For patients with LPBS, the addition of R-E-NSM does not increase the incidence of complications and is not a risk factor.

Since complication rates fluctuate over time between the initial surgery and subsequent R-E-NSM, we determined a 62-day cut-off for complications using the ROC curve. The median operation time of the IBS group was approximately 13.5 minutes longer than that of the NPBS group; this difference may be due to the IBS group requiring removal of the nipple during surgery and nipple reconstruction as well. Additionally, the distributions of Ki-67 and PR positivity and the rate of neoadjuvant chemotherapy use varied across groups. Previous studies have suggested that tumor downstaging after neoadjuvant chemotherapy may provide surgical opportunities for some breast cancer patients (29, 30); while the proportion of neoadjuvant chemotherapy in the IBS group was the lowest, this might indicate that their tumor burden was relatively minor. Regarding incision-related features, among 220 patients who had intraoperative breast surgery or a history of PBS, periareolar arc incisions made up 59.1%, indicating that in prior breast surgeries, this type of incision was chosen for both tumor exposure and aesthetic considerations (31).

In the unadjusted analysis, patients with SPBS appeared to have higher crude rates of any complications (28.6% vs. 22.0%), minor complications (25.0% vs. 20.9%), and CDC ≥ 2 complications (23.2% vs. 17.0%) than those in the NPBS group. The wound dehiscence (reoperation) rate in the SPBS group was 3.6%, compared with 0% in the LPBS group and 0.6% in the NPBS group. However, after adjusting for baseline differences including age, tumor stage, neoadjuvant chemotherapy, operation time, operation type, and follow-up duration in the mixed-effects regression model, SPBS was no longer significantly associated with increased complication risk (any complications: OR 1.579; 95% CI 0.791–3.155; P = 0.195). This discrepancy between crude and adjusted analyses suggests that the higher observed complication rates in the SPBS group may be partially attributable to confounding factors, such as operation type and more advanced disease requiring longer operations, rather than to the short surgical interval per se. Several methodological considerations may also contribute to this non-significant finding. First, the relatively small sample size of the SPBS group (n=56) limited statistical power, resulting in a wide confidence interval that reflects substantial uncertainty around the effect estimate. Second, the number of outcome events was modest, further reducing the precision of the estimate. Third, the inclusion of multiple covariates in the mixed-effects model may have led to overfitting, potentially diluting the apparent association. Although a non-significant trend toward increased odds was observed, clinicians should exercise caution when planning R-E-NSM for patients with short-term surgical history (≤62 days). Larger, adequately powered studies are needed to clarify whether SPBS truly increases the risk of complications after R-E-NSM. Among SPBS patients, implant loss occurred in 3.6% of cases, which remains comparable to or lower than rates reported for open surgery (32). Previous studies have shown that the failure rate of breast reconstruction after mastectomy ranges from 2.9% to 9% (33–36). Moreover, the incidence of major complications in the SPBS group (5.4%) was comparable to that of major complications in breast reconstruction after prior mastectomy (4.0% ~ 7.1%) (37–39), suggesting that R-E-NSM surgery is a safe alternative, even when considering a short-term surgical history.

Conversely, we observed that LPBS was associated with lower complication rates. The incision usually heals completely, with minimal impact on the incision flap and the formation of recanalized blood vessels, which helps reduce the risk of surgical complications. Although there was no significant difference in oncological safety among the four groups, we hypothesized that when a long interval has passed since PBS, surgeons—concerned about blood flow and hidden foci within scarred tissue—often leave a thicker skin flap during R-E-NSM, decreasing immediate complications but possibly increasing the chance of microscopic residual disease. While this finding was statistically significant in the mixed-effects model, we acknowledge that the smaller sample size of the LPBS group (n=75) and potential unmeasured confounders may contribute to this observation. Therefore, we conclude that the LPBS group has lower surgical complications and is not a risk factor for surgery, rather than indicating a definitive protective effect, and caution is warranted when interpreting these results as causal, larger studies with longer follow-up are needed to verify this hypothesis.

In R-E-NSM surgery, some patients may need the removal of the NAC, the skin over the tumor, or dilation of a preoperative incision, which we categorized as IBS. In open surgery, blood supply to the incision flap was compromised due to prolonged traction and an extended incision. In contrast, R-E-NSM surgery involved dilating the preoperative incision to create a fresh margin that could be sutured directly (3, 28). Although some incisions were preserved on parts of the breast, such as the NAC and the skin over the tumor, R-E-NSM surgery moved the incision to the armpit, thereby minimizing the extent of removal and reducing tension at the incision site. As a result, we concluded that IBS did not increase the risk of complications. In fact, complication rates were significantly lower than those seen with open surgery, suggesting that R-E-NSM surgery provides a safer option profile (28, 32).

We examined how incision-related factors affect surgical complications and found that incision position, side, and length had little effect. Our analysis showed that complications were mainly linked to the time gap between previous incisions rather than to incision-specific factors. As a result, more focus should be placed on the interval between surgeries. Additionally, the high rate of missing data on incisions (14.1%) could affect the findings, underscoring the need for a larger sample size in future research.

We prospectively evaluated the association of IBS, SPBS, LPBS, and NPBS with Breast-Q and Ueda scores. We found that, for women undergoing breast reconstruction, there was no significant difference in Breast-Q scores among the four groups. BREAST-Q focuses on subjective experiences (satisfaction, pain, emotional impact, and sexual life effects) (25, 40). The overall experience has a greater influence on patients’ satisfaction than any single indicator. Therefore, there is no significant statistical difference in BREAST-Q scores among the four groups. The Ueda score focuses more on objective anatomical measures, such as symmetry, scar quality, and contour shape (26). In the NPBS group, since there was no previous incision and the anatomical landmarks were intact, the “excellent and good rate” of the early Ueda score was the highest. Moreover, the difference in Ueda scores was limited to 1–3 months after surgery and disappeared by 6 months. We believe that as postoperative edema and incision-site pigmentation gradually resolve, the objective aesthetic scores of the SPBS, LPBS, and IBS groups converge with those of the NPBS group over time. Furthermore, we used mixed-effects modeling to assess the impact of PBS on Ueda scores. First, in the IBS group, simultaneous tumor surface incision, nipple resection, and reconstruction or extended incision were performed during the operation; this theoretically increased the difficulty of achieving symmetry. Although this study showed that the early Ueda score was lower in this group, the BREAST-Q score was the same as that of the other groups. It might be because the immediate completion of nipple reconstruction avoided the psychological shock of the patient undergoing another surgery. Second, we found that at 3 months postoperatively, the SPBS group had significantly lower aesthetic scores than the NPBS group, whereas differences for the LPBS and IBS groups were not statistically significant. This suggests that scarring and residual inflammation from recent biopsies or incisions temporarily diminish early aesthetic satisfaction. Lastly, the absence of any significant difference in postoperative Ueda scores between the LPBS and NPBS groups likely reflects the long interval since the previous operation: the original scar area has fully matured, so the “visibility of scar” item on the Ueda scale is not penalized, and collagen remodeling plus neovascularization have restored flap elasticity to near-baseline levels.

The advantages of this study are as follows. First, this is the first study to compare previous breast surgeries with postoperative surgical complications and aesthetic outcomes of R-E-NSM. Second, it simultaneously evaluated surgical complications, patient-reported outcomes (BREAST-Q), physician-reported aesthetics (Ueda score), and oncologic outcomes, providing a comprehensive assessment of surgical safety and efficacy. Lastly, this study enrolled 1, 081 patients and prospectively collected data, employing multivariable mixed-effects regression models to enhance the reliability of causal inference. Although our findings are novel, there are limitations. First, for the patient samples with previous breast surgery, the sample size is relatively small, which may lead to errors in the results. Second, oncologic outcomes still need validation with larger sample sizes and longer follow-up. Finally, our analysis is based on data from a single institution where experienced surgeons managed a high annual caseload. Therefore, the applicability of our findings may be limited to institutions with similar caseloads and expertise.

## 5 Conclusions

LSPB (>62 days) is not a risk factor for R-E-NSM. However, for patients with a SPBS (≤62 days), although the risk does not increase significantly after adjustment, caution is warranted when performing R-E-NSM.

## Data Availability

The raw data supporting the conclusions of this article will be made available by the authors, without undue reservation.
