# Supplementary material for: Evaluating the impacts of previous breast surgery on surgical complications and aesthetic outcomes in reverse-sequence endoscopic-assisted nipple-sparing mastectomy with or without direct-to-implant breast reconstruction
Source: Front Oncol. 2026 Sep 9;16:1860773. doi: 10.3389/fonc.2026.1860773 (PMC13597230; doi:10.3389/fonc.2026.1860773)
Supplement: Supplementary file 1 [file DataSheet1.docx]

**Supplementary Materials - Index**

| **Supplementary Figures and Tables** |  |
| --- | --- |
| Supplementary Figure 1 |  |
| Supplementary Figure 2 |  |
| Supplementary Figure 3 |  |
| Supplementary Table 1 |  |
| Supplementary Table 2 |  |
| Supplementary Table 3 |  |
| Supplementary Table 4 |  |

**Supplementary Figures and Tables**

**Supplementary Figure 1.** Flow Chart of Participant Selection

R-E-NSM, reverse-sequence endoscopy-assisted nipple-sparing mastectomy; MMT, mammotome.

**Supplementary Figure 2.** The ROC curve of the relationship between previous breast surgery time and complications

ROC, receiver operating characteristic; CDC, Clavien–Dindo classification; SSI, surgical site infection; AUC, area under the curve.

The optimal cutoff of 62 days was determined by ROC curve analysis based on the occurrence of any complications, using the Youden index to maximize both sensitivity and specificity.

**Supplementary Figure 3.** Kaplan–Meier survival curve of oncologic outcomes in the NPBS, IBS, SPBS, and LPBS groups.

(a) The probability of LRFS between four groups; (b) The probability of DMFS between four groups; (c) The probability of DFS between four groups.

IBS, intraoperative breast surgery; SPBS, short-term previous breast surgery; LPBS, long-term previous breast surgery; NPBS, no previous breast surgery; LRFS, local recurrence-free survival; DMFS, distant metastasis-free survival; DFS, disease-free survival.

**Supplementary Table 1.** Incision-Related Features of 220 Patients in the IBS, SPBS, and LPBS Groups

| Characteristic | IBS(n=89) | SPBS(n=56) | LPBS(n=75) | P value |
| --- | --- | --- | --- | --- |
| Incision location |  |  |  | <0.001 |
| NA | 16 (18.0) | 7 (12.5) | 8 (10.7) |  |
| Periareolar arc incision | 72 (80.9) | 23 (41.1) | 35 (46.7) |  |
| Any incision on the dome of the breast | 0 (0.0) | 20 (35.7) | 21 (28.0) |  |
| Inferior fold incision | 0 (0.0) | 0 (0.0) | 2 (2.7) |  |
| Lateral radial incision | 1 (1.1) | 6 (10.7) | 9 (12.0) |  |
| Laterality |  |  |  | 0.384 |
| NA | 16 (18.0) | 7 (12.5) | 8 (10.7) |  |
| Left | 39 (43.8) | 22 (39.3) | 39 (52.0) |  |
| Right | 34 (38.2) | 27 (48.2) | 27 (36.0) |  |
| Bilateral | 0 (0.0) | 0 (0.0) | 1 (1.3) |  |
| Incision length, cm, M (Q_1_, Q_3_) | 2.0 (2.0, 3.0) | 3.0 (2.0, 4.0) | 3.0 (2.0, 3.5) | 0.010 |

IBS, intraoperative breast surgery; SPBS, short-term previous breast surgery; LPBS, long-term previous breast surgery; NPBS, no previous breast surgery; M, median; NA, not available.

**Supplementary Table 2.** Mixed-Effect Regression Model of the Relationship Between Incision-Related Features and Surgical Complications

|  | Any complication | | Major complication | | Minor complication | | CDC≥2 | | Repeated SSI | |
| --- | --- | --- | --- | --- | --- | --- | --- | --- | --- | --- |
|  | OR | P | OR | P | OR | P | OR | P | OR | P |
| NPBS | Ref | NA | Ref | NA | Ref | NA | Ref | NA | Ref | NA |
| Incision location |  |  |  |  |  |  |  |  |  |  |
| Periareolar arc incision | 0.702(0.343-1.438) | 0.334 | 0.910(0.242-3.424) | 0.889 | 0.759(0.369-1.559) | 0.452 | 0.854(0.401-1.817) | 0.681 | 1.122(0.466-2.701) | 0.797 |
| Any incision on the surface of the breast | 1.015(0.449-2.296) | 0.971 | 1.015(0.211-4.877) | 0.986 | 0.936(0.401-2.184) | 0.878 | 1.184(0.507-2.766) | 0.696 | 1.082(0.368-3.180) | 0.886 |
| Inferior fold incision | 2.046E-7(0.000) | 0.996 | 0.967(0.000-4156.050) | 0.994 | 7.630E-8(0.000) | 0.997 | 0.131(3.870E-5-444.190) | 0.624 | 0.464(0.000-1771.492) | 0.855 |
| Lateral radial incision | 0.272(0.032-2.340) | 0.235 | 1.719(0.219-13.477) | 0.606 | 1.263E-7(0.000) | 0.992 | 0.414(0.058-2.976) | 0.381 | 0.318(0.020-5.064) | 0.417 |
| Laterality |  |  |  |  |  |  |  |  |  |  |
| Left | 0.585(0.299-1.142) | 0.116 | 0.927(0.294-2.921) | 0.896 | 0.606(0.306-1.201) | 0.151 | 0.592(0.287-1.223) | 0.157 | 0.525(0.202-1.362) | 0.185 |
| Right | 0.948(0.488-1.842) | 0.874 | 1.162(0.364-3.708) | 0.800 | 0.840(0.418-1.685) | 0.623 | 1.341(0.679-2.646) | 0.398 | 1.727(0.784-3.801) | 0.175 |
| Bilateral | 4.094E-7(0.000) | 0.997 | 1.414(1017E-5-196629.586) | 0.954 | 4112E-7(0.000) | 0.997 | 0.296(2.284E-6-38421.917) | 0.839 | 0.775(5.840E-6-102962.520) | 0.966 |
| Incision length |  |  |  |  |  |  |  |  |  |  |
| ≤3 cm | 0.683(0.380-1.230) | 0.204 | 1.003(0.359-2.806) | 0.995 | 0.651(0.353-1.200) | 0.168 | 0.901(0.490-1.657) | 0.738 | 1.241(0.611-2.522) | 0.549 |
| ＞3 cm | 0.886(0.382-2.053) | 0.778 | 1.125(0.263-4.806) | 0.874 | 0.863(0.362-2.056) | 0.739 | 0.798(0.318-2.002) | 0.630 | 0.353(0.078-1.611) | 0.179 |

SSI, surgical site infection; NPBS, No previous breast surgery; Ref, reference; NA, not available; OR, odds ratios; CI, confidence intervals.

Footnotes: Mixed-Effect Regression Model adjusted OR (95%CI): adjust for age, BMI, cup, breast ptosis, pathologic T stage, pathologic N stage, neoadjuvant chemotherapy, operation time, operation type, laterality, axillary lymph node management, nipple resection, nipple reconstruction, adjuvant chemotherapy, radiotherapy.

**Supplementary Table 3.** Mixed-Effect Regression Model of Postoperative 6 Months Patient-Reported Outcomes on BREAST-Q Score in the IBS, SPBS, LPBS, NPBS Groups

| Variable | Satisfaction with breast a | Psychological well-being a | Physical well-being a | Sexual well-being a |
| --- | --- | --- | --- | --- |
| NPBS | Ref | Ref | Ref | Ref |
| IBS | -1.046 (-18.381 - 5.621) | -0.910 (-21.739 - 7.993) | 0.680 (-8.757 - 17.998) | -0.556 (-21.155 - 11.833) |
| SPBS | 0.589 (-5.884 - 10.907) | 0.805 (-6.147 - 14.651) | -0.920 (-13.740 - 4.989) | 1.199 (-4.608 - 18.949) |
| LPBS | 0.492 (-6.367 - 10.611) | -0.174 (-11.450 - 9.589) | -0.276 (-10.794 - 8.138) | -0.307 (-14.290 - 10.439) |

IBS, intraoperative breast surgery; SPBS, short-term previous breast surgery; LPBS, long-term previous breast surgery; NPBS, no previous breast surgery; Ref, reference.

^a^ Postoperative 6 months BREAST-Q score in satisfaction with breast NA=670, postoperative 6 months BREAST-Q score in psychosocial well-being NA=668, postoperative 6 months BREAST-Q score in physical well-being NA=670, postoperative 6 months BREAST-Q score in sexual well-being NA=692, seven patients have no sexual life.

* P<0.05

Footnotes: Mixed-Effect Regression Model adjusted OR (95%CI): adjust for age, BMI, cup, breast ptosis, pathologic T stage, pathologic N stage, neoadjuvant chemotherapy, operation time, operation type, laterality, axillary lymph node management, nipple resection, nipple reconstruction, adjuvant chemotherapy, radiotherapy.

**Supplementary Table 4.** Mixed-Effect Regression Model of Postoperative Physician-Reported Outcomes on Ueda Score in the IBS, SPBS, LPBS, NPBS Groups

| Variable | Postoperative 1-month a | Postoperative 3-month a | Postoperative 6-month a |
| --- | --- | --- | --- |
| NPBS | Ref | Ref | Ref |
| IBS | -1.514 (-3.990 - 0.515) | -0.988 (-2.800 – 0.926) | 0.647 (-4.008 – 7.943) |
| SPBS | -1.639 (-1.569 - 0.141) | -2.457 (-1.880 - -0.210)* | 0.114 (-1.127 - 1.266) |
| LPBS | 0.486 (-0.712 - 1.182) | 1.428 (-0.320 - 2.030) | 1.022 (-0.509 - 1.614) |

IBS, intraoperative breast surgery; SPBS, short-term previous breast surgery; LPBS, long-term previous breast surgery; NPBS, no previous breast surgery; Ref, reference.

^a^ Ueda in postoperative 1 month NA=221, Ueda in postoperative 3 months NA=421, Ueda in postoperative 6 months NA=548.

* P<0.05

Footnotes: Mixed-Effect Regression Model adjusted OR (95%CI): adjust for age, BMI, cup, breast ptosis, pathologic T stage, pathologic N stage, neoadjuvant chemotherapy, operation time, operation type, laterality, axillary lymph node management, nipple resection, nipple reconstruction, adjuvant chemotherapy, radiotherapy.
